# Using a Mobile App to Support Parents of Children with Behavior Problems

**DOI:** 10.1007/s10802-025-01385-z

**Published:** 2025-10-30

**Authors:** Angela V. Dahiya, Rosanna Breaux, Stephanie N. Pham, Daniele C. Martino, Megan Fok, Jordan Albright, Delshad M. Shroff, Angela Scarpa

**Affiliations:** 1 Department of Psychology, Virginia Tech, 460 Turner St. NW, Suite 207, Blacksburg, VA 24060 USA; 2 Virginia Tech Autism Clinic & Center for Autism Research, Blacksburg, VA USA; 3 Virginia Tech Child Study Center, Blacksburg, VA USA; 4 Department of Psychiatry, Kaiser Permanente ASD Center, San Jose, CA USA; 5 Children’s Hospital of Philadelphia, Philadelphia, PA USA; 6 Department of Psychology, University of South Alabama, Mobile, AL USA; 7 Virginia Treatment Center for Children, VCU Health, Richmond, VA USA

**Keywords:** Behavioral parent training, Telehealth, mHealth, Autism, ADHD, Disruptive behaviors

## Abstract

Evidence-based mental health services are difficult to access; telehealth and mobile health hold promise by removing barriers to traditional clinic-based interventions and enabling broader access. Behavioral parent training (BPT) is an evidence-based treatment for child disruptive behaviors. This project examined the feasibility, acceptability, and preliminary efficacy of using a mobile BPT app, *Treks*, with families as a standalone treatment (Study 1) or in combination with brief clinician consultation (Study 2). Study 1 participants included 20 caregivers of children with challenging behaviors who engaged with *Treks* for four weeks. Study 2 participants included 26 caregivers of autistic children with behavioral concerns; all parents received a one-session telehealth consultation followed by random assignment to four weeks of *Treks* engagement (Treks; *n* = 14) or access to online resources (consultation control: CC; *n* = 12). Across both studies, *Treks* was rated positively and was reported by parents as acceptable and appropriate for their concerns, as well as adequately feasible. In Study 1, 60% of participants fully completed *Treks* and 83% of completers showed reliable improvement in at least one main outcome (parenting stress, parent sense of competence, and child behavior problems). In study 2, there were significant improvements in Treks but not CC participants, showing moderate-to-large decreases in child disruptive behaviors and parent stress and increases in parent-perceived competency. Mobile app-delivered BPT has the capacity to support families as a feasible and acceptable standalone treatment and should be considered as part of a stepped-care approach or for families who cannot access clinician-delivered BPT.

Despite rising rates of youth mental and behavioral health problems, evidence-based mental health services remain difficult to access (Chien et al., 2022), particularly for families in rural areas. This results in rural youth experiencing fewer positive outcomes than their urban and suburban counterparts (Mello et al., 2016). Telehealth (i.e., the delivery of services using information and communication technology) has been identified as one solution to overcome access barriers related to cost, distance, and provider availability (Ashburner et al., 2016). Telehealth and mobile health interventions hold promise for improving mental health parity for youth by removing barriers to traditional clinic-based interventions and enabling access for a broader range of families (see Breaux et al., 2023; Ellison et al., 2021). Behavioral parent training (BPT) is a first-line, evidence-based treatment for disruptive behavior problems among children ages 2–12 years (Gresham, 2015) that has been linked to short and long-term benefits for children, caregivers, families, and society (see Lundahl et al., 2006; McCart et al., 2006; Rimestad et al., 2019). BPT has been used transdiagnostically across childhood disorders such as autism spectrum disorder (ASD), attention deficit/hyperactivity disorder (ADHD), disruptive mood dysregulation disorder, intermittent explosive disorder, oppositional defiant disorder (ODD), and conduct disorder (Gresham, 2015). There is limited research exploring mobile app delivered BPT as an enhancement to traditional BPT (Parent et al., 2022), or as a standalone intervention (Feil et al., 2018; Lee et al., 2024). As such, the current study sought to examine the feasibility, acceptability, and preliminary efficacy of using a mobile app delivered BPT intervention to families as a standalone treatment (Study 1; transdiagnostic) or in combination with brief clinician consultation (Study 2; focused on ASD).

## Behavioral Parent Training for Child Behavior Problems

A range of BPT programs exist, with Defiant Children (Barkley, 2013), Incredible Years (Webster-Stratton, 2000), Parent–Child Interaction Therapy (PCIT; Eyberg & Funderburk, 2011), and The Positive Parenting Program (Triple P; Sanders, 1999) being some of the most widely implemented programs. Across BPT programs, sessions generally cover the same psychoeducational and behavioral principles, though the specific orders and strategies vary (see Kaehler et al., 2016 for a review). For example, using the Antecedent-Behavior-Consequence (ABC) model, parents are taught how to conduct a functional behavioral analysis to understand the triggers and situations that precede a specific behavioral problem as well as prevention and reinforcement strategies. Additionally, programs also include opportunities for practicing specific strategies (e.g., prevention strategies, reinforcement, planned ignoring) to increase parental attention to positive/desired behaviors and to address problematic (e.g., aggressive) and non-compliance behaviors (Johnson et al., 2019).

Although BPT is a first-line treatment for behavior problems and has been used with a range of clinical populations, there is also a history of research focusing on the use of BPT for autistic children specifically. These autism specific BPT programs generally include didactic material about behavioral problems in autistic children in addition to information on ASD and general behavior management training (Postorino et al., 2017; Reitzel et al., 2013; Sofronoff et al., 2004). For example, The RUBI Autism Network’s Parent Training Manual for Disruptive Behaviors (Bearss et al., 2018a, b), which was developed for use with parents of autistic children ages 3 to 7 years displaying problematic behaviors, has been delivered in person and via telehealth (Bearss et al., 2015a, b, 2018a, b). Similarly, the COMPASS for Hope intervention (Kuravackel et al., 2018) is an 8-week program for parents of autistic children between the ages of 3 to 12 years that has been delivered in person and via telehealth using a combination of group and individual sessions, and has been found to be useful for rural families (Dahiya et al., 2022).

## Mobile Applications (Apps) for Child Behavior Problems

A recent systematic review concluded that using telehealth is equivalent to or even better than services provided in-person for reducing child behavior problems (Ellison et al., 2021), including parent-facilitated or parent-mediated interventions conducted via videoconferencing. Moreover, mobile apps may be an especially promising medium of accessible treatment delivery given the higher rates of mobile phone use among low-income families compared to high-income families (Blumberg & Luke, 2018). Technology-enhanced BPT has been found to increase treatment accessibility, facilitate higher treatment engagement and skill acquisition, and improve child behavior (Baumel et al., 2017; Parent et al., 2022).

Despite the widespread use of parenting apps in recent decades, a recent systematic review highlighted that none of these apps teach all six of the core evidence-based BPT techniques, with ~ 75% teaching two or fewer evidence-based techniques (Mandelbaum et al., 2024). Use of effective commands was included in the fewest number of applications (i.e., 4%). Additionally, despite research demonstrating the potential of mobile apps as enhancements to clinician-delivered BPT, limited research exists on the implementation of mobile apps for BPT as a potential standalone treatment. Specifically, Feil et al. (2018) conducted feasibility testing of the ParentNet app with 73 parents and found high satisfaction and utilization, and small to moderate improvements in parenting behavior. Additionally, Lee et al. (2024) conducted a randomized controlled trial (RCT) examining the utility of a mobile app-assisted BPT program for reducing behavior problems in autistic children ages 3–7 years in Korea. Their study demonstrated the feasibility of using mobile apps to deliver BPT and reduce behavior problems in young autistic children. Together, these few studies provide initial promise for the potential of a mobile BPT app as a feasible and accessible treatment to address behavior problems in children; however, more research on a broader age range of children and assessment of clinically meaningful improvements in child and parent outcomes is critically needed.

### The Treks App

*Treks* (https://www.th.ru.st/treks; Thrust Interactive, Inc.) is a behavior change mobile app that supports users through a game that helps them create small habits that can grow into lifestyle changes. The game incorporates technological support, such as instructional videos, strategies to attempt, options of activities to commit to and engage in, and reinforcement for effort. For the current study, *Treks* was used as the foundation for teaching evidence-based BPT principles to aid parents in learning and consistently using behavioral strategies aimed at reducing their child’s challenging behaviors and increasing parental confidence in managing behaviors. We customized *Treks* to consist of 28 brief daily lessons completed over the course of a month (see Table 1). The principles used in our *Treks* app included evidence-based principles of behavior modification: the ABC model of behavior, prevention strategies, visual schedules, reinforcement, planned ignoring, replacement behaviors, compliance training, teaching techniques, and generalization and maintenance of behaviors.

**Table 1:** Treks app content

Trek session	Study 1: Transdiagnostic	Study 2: Autism
1	Behavior Principles	Challenges in Parenting
2	The ABCs of Behavior: Part 1	The ABCs of Behavior: Part 1
3	The ABCs of Behavior: Part 2	The ABCs of Behavior: Part 2
4	Tracking Your Child’s Behaviors	Tracking Your Child’s Behaviors
5	Results of the ABC Chart	Results of the ABC Chart
6	Recap of the ABCs	Recap of the ABCs
7	Parent Mental Health Day	Parent Mental Health Day
8	Helping Your Child Deal with Change	Helping Your Child Deal with Change
9	Prevention Strategies: Part 1	Prevention Strategies: Part 1
10	Prevention Strategies: Part 2	Prevention Strategies: Part 2
11	Prevention Strategies: Part 3	Prevention Strategies: Part 3
12	How to Make a Visual Schedule	How to Make a Visual Schedule
13	Making Your Own Visual Schedule	Making Your Own Visual Schedule
14	Parent Mental Health Day	Parent Mental Health Day
15	Types of Reinforcements	Types of Reinforcements
16	How to Identify Reinforcements	How to Identify Reinforcements
17	Reinforcing Behaviors	Reinforcing Behaviors
18	Setting Up a Point/Token System	Planned Ignoring
19	Implementing a Point/Token System	Practice Ignoring
20	Planned Ignoring	Using Replacement Behaviors
21	Practice Ignoring/Parent Mental Health Day	Parent Mental Health Day
22	Using Replacement Behaviors	Teaching Compliance
23	Teaching Compliance	Practicing Compliance Training
24	Practicing Compliance Training	Teaching New Skills
25	Extending Compliance Training to School	Teaching Techniques
26	Teaching Coping Skills	Time to Teach!
27	Generalization & Maintenance	Generalization & Maintenance
28	Planning for the Future	Planning for the Future

Although most of the *Treks* topics are the same, we did update some videos so that the ones for the transdiagnostic study referred to behaviors in general, and the ones for the Autism study were more autism-specific

### Present Study

With this backdrop in mind, the overarching goal of the current study was to conduct pilot studies of the *Treks* BPT mobile app to provide a cost-effective, evidence-based program that could be used either as a standalone treatment for families in rural or other underserved communities, or as a supplement to consultations with clinicians to improve challenging child behaviors. This goal was carried out through two studies: a pilot feasibility trial implementing *Treks* transdiagnostically (Study 1), and a pilot RCT examining its use in combination with consultation sessions for families of autistic children (Study 2). The primary aim of Study 1 was to assess the feasibility and efficacy of the *Treks* app by collecting mixed-methods caregiver feedback on the acceptability, usability, and benefits of *Treks* in terms of child behavior problems, parenting stress, and parental sense of competency, and assessing the percentage of families of children with behavior problems who complete the entire *Treks* app. The specific aims of Study 2 were to investigate (1) feasibility of the *Treks* mobile app by examining attrition, compliance, ease of use, and appropriateness, and (2) benefits on parent and child outcomes following use of the app over the course of 4 weeks as an enhancement to a telehealth consultation versus a consultation control condition with access to online resources over the course of 4 weeks. It was hypothesized that the *Treks*-supplemented consultation model (Treks group) would be feasible, evidenced by high compliance, and high parental ratings of satisfaction, appropriateness, acceptability, and feasibility. It was further hypothesized that the Treks group would reveal greater improvements in parent competence and stress compared to the consultation control (CC) group.

## Methods

### Study 1 – Transdiagnostic Feasibility Pilot Study

#### Participants and Procedures

Participants included 20 parents (85% mothers; aged 31–59 years; *M* = 40.85, *SD* = 0.294) of children ages 2–12 years (*M* = 6.15, *SD* = 0.294) with challenging behaviors residing in rural Appalachia. Participants did not need to have a formal mental health diagnosis to be enrolled, and in fact only six children (30.0%) had prior diagnoses (all either ADHD, ASD, and/or ODD) as participants were recruited largely from clinic waitlists with many families waiting for a comprehensive autism or psychoeducational assessment. As can be seen in Table 2, the sample represented predominately middle-to-upper class socioeconomic statuses based on family income and parental education. Participants were allowed to be enrolled in treatment with stable medication or no medication prior to entry and were not asked to stop services while enrolled in the study. Eligible families were screened over the phone or via email, prior to being provided with an online consent form providing more information about the study. Eligible families who consented to participate were then sent information regarding accessing the *Treks* BPT mobile app.

**Table 2:** Demographic information for both studies

Demographic variable	Study 1	Study 2		
	*(N* = 20)	Treks (*n* = 14)	CC (*n* = 12)	
	*M(SD)*	*M (SD)*	*M (SD)*	*t*
Child Age	6.15(0.29)	6.50 (2.85)	7.17 (3.07)	-0.57
Parent Age	40.85(0.29)	40.64 (6.20)	37.42 (5.52)	1.39
Child Receptive Language Ability –		87.77 (16.14)	95.25 (25.11)	-0.89
	***N (%)***	***N (%)***	***N (%)***	**χ**^**2**^
Family Income				2.00
$25 k to $50 k	2 (10)	1 (7)	3 (25)	
$50 k to $75 k	3 (15)	4 (29)	2 (17)	
$75 k to $100 k	4 (20)	3 (21)	3 (25)	
$100 k to $200 k	10 (50)	2 (14)	1 (8)	
Above $200 k	1 (5)	4 (29)	3 (25)	
Child Sex Assigned at Birth				0.35
Male	12 (60)	9 (64.3)	9 (75)	
Female	8 (30)	5 (35.7)	3 (25)	
Parent Sex Assigned at Birth				0.01
Male	3 (15)	1 (7)	1 (8)	
Female	17 (85)	13 (93)	11 (92)	
Parent Race/Ethnicity				5.57
White	18 (90)	7 (50)	11 (92)	
Black	0 (0)	0 (0)	0 (0)	
Asian	0 (0)	4 (29)	1 (8)	
Latine	0 (0)	0 (0)	0 (0)	
Biracial or Multiracial	2 (10)	3 (21)	0 (0)	
Parent Education				8.99
High School	2 (10)	0 (0)	1 (8)	
Trade/Vocational Training	1 (5)	4 (29)	0 (0)	
Assoc. Degree	0 (0)	0 (0)	2 (17)	
College Graduate	9 (45)	4 (29)	1 (8)	
Graduate School	8 (40)	6 (42)	8 (67)	
Child Medication Status				
For ADHD	3 (15)	4 (29)	3 (25)	1.17
For other difficulties	0 (0)	4 (29)	5 (42)	0.12

*CC* Consultation Control condition; *ADHD* Attention-deficit/hyperactivity disorder. All *p*s > .062 for *t*-tests and **χ**^**2**^ tests comparing the Treks and CC conditions for Study 2. The Peabody Picture Vocabulary Test, 5th Edition was used to measure receptive language ability. The raw score was calculated by subtracting the number of errors the child makes from a total ceiling score, which is then converted to a standard score (*M* = 100, *SD* = 15)

Although *Treks* BPT was intended to be completed over a one-month period, the majority of participants needed longer, as they did not complete a lesson each day. Specifically, the Treks BPT program was designed as a 28-day program, and families were advised to self-pace their interaction with the app within a 30-day timeframe. Outcome measures (i.e., parenting stress, parent sense of competence, and child behavior problems) were completed at pre- and post-completion of the program (Time 1 and Time 2, respectively). Measures of usability, acceptability, appropriateness, and feasibility were collected at Time 2. Data were stored on Research Electronic Data Capture (REDCap; Harris et al., 2009). Participants were compensated for completing study measures at each timepoint. This study was approved by the Virginia Tech Institutional Review Board and was performed in accordance with the ethical standards laid down in the 1964 Declaration of Helsinki.

### Measures

#### Parenting Stress Index, 4.^th^ Edition, Short Form (PSI-4-SF; Abidin, 2012)

The PSI-4 is a 36-item scale that measures the amount of stress parents experience in their parenting role within the domains of child characteristics, parent characteristics, and the parent–child relationship. The items are rated on a 5-point Likert scale ranging from *Strongly Disagree* to *Strongly Agree*. The PSI demonstrates strong internal consistency (α ≥ .96 for each domain and test–retest reliability ranging from .65 to .96 for the Total Stress score; Abidin, 2012). In the current study, Cronbach’s alpha indicated good and excellent internal consistency at each timepoint, respectively (α_T1_ = .83, α_T2_ = .94).

#### Parenting Sense of Competence Scale (PSOC; Johnston & Mash, 1989)

The PSOC is a 16-item scale that assesses parental confidence in regard to how they feel they can handle their child’s problem behaviors. The items are rated on a 6-point Likert scale (ranging from *Strongly Disagree* to *Strongly Agree*). The Cronbach’s alpha indicated fair internal consistency at both timepoints for the total score (*α*_*T1*_ = .55, *α*_*T2*_ = .72).

#### Eyberg Child Behavior Inventory (ECBI; Eyberg & Pincus, 1999)

The ECBI is a 36-item scale of problem behavior in children, consisting of two scales: Intensity and Problem. We used the Intensity scale, which measures how frequently each disruptive behavior occurs on a 7-point scale ranging from *Never* to *Always,* with positive scores indicating higher frequency of problem behaviors. The ECBI’s test–retest reliability (> 0.75) and internal consistency (> 0.90) are high (Funderburk et al., 2003). Good and excellent internal consistency was found in this study at each timepoint, respectively (α_*T1*_ = .88 α_*T2*_ = .95).

#### Acceptability, Appropriateness, and Feasibility of Implementation

Acceptability, appropriateness, and feasibility was measured using the *Acceptability of Intervention Measure* (AIM), *Intervention Appropriateness Measure* (IAM), and *Feasibility of Intervention Measure* (FIM; Weiner et al., 2017). These measures include 4 items each and have been shown to be indicators of implementation success. Items are rated on a 5-point scale ranging from *Completely Disagree* to *Completely Agree*. These scales demonstrate strong psychometric properties in terms of content validity, discriminant content validity, reliability, structural validity, structural invariance, known-groups validity, and responsiveness to change (Weiner et al., 2017). In the current study, internal consistency was good to excellent for the AIM (α = .93), IAM (α = .94), and FIM (α = .88).

### Data Analytic Plan

Descriptive statistics were used to assess completion rates. To test the hypothesis of acceptability, appropriateness, and feasibility of the app, scores from the AIM, IAM, and FIM were calculated to determine an overall mean score, which demonstrates the extent to which the *Treks* app is judged as suitable for parents of children with behavioral problems. An average score of 3 or higher on each item indicates adequate acceptability, appropriateness, and feasibility. To examine preliminary evidence of efficacy for the *Treks* mobile app, we conducted paired samples *t*-test using R programming language (packages: tidyverse, haven, ggplot2) to assess change from pre- to post-*Treks* in child behavior problems, parent stress level, and parent self-perceived competence. Individual level change in these three constructs was also examined using calculations of reliable change index (RCI; Jacobson & Truax, 1991), which evaluates the change over time of an individual measurement score to determine meaningful change. RCI is calculated as a ratio of the observed difference score to standard error of measurement of the difference. RCI values ± 1.96 are recommended to infer statistically significant and meaningful change. Additionally, given the small sample size of this pilot study, effect sizes were examined in addition to significance levels and were the primary focus of interpretation for efficacy.

## Results

Sixty percent of participants who consented to the study completed the entire *Treks* BPT mobile app; 12 parents completed both Time 1 and 2 outcome measures. Parents who completed the study vs. those who did not did not significantly differ any key demographic (e.g., child and parent age, parent education) or clinical (e.g., child behavior problems, parent sense of competency) variables, |*t|*= 0.02–1.51 and χ^2^ = .04—1.20, *p* > .085 (with the one marginally significant finding being parents with lower parenting stress at Time 1 were marginally less likely to complete the study). Scores from the AIM, IAM, and FIM questionnaires indicated that the *Treks* BPT app was acceptable (*M* = 3.29, *SD* = 0.88, *Range* = 2–5), appropriate (*M* = 3.23, *SD* = 0.54 *Range* = 2–4), and feasible (*M* = 3.19, *SD* = 0.70 *Range* = 2–4) for addressing parenting for children with transdiagnostic behavioral concerns on average, though some families did report less than adequate scores. Qualitative feedback for improvement from parents via open-ended questions indicated that it was difficult to find time to engage with the app, that parents wanted more flexibility in and reminders for utilizing the app, and that parents would like there to be a log or way to check their prior responses in the app. Some participants also reported that they experienced unexpected life obstacles and/or technical issues that posed as barriers to engaging fully with the app content. This qualitative feedback is organized in Table 3 along with exemplar quotes.

**Table 3:** Qualitative feedback for areas of improvement from Study 1

Topic of feedback	Example feedback
Lack of Reminders for Engagement	*"It would have been nice if there were a reminder for each day so I could have done more of the app."* *"There was no suggestion as to what time of day would be best to use the Treks app; it would be nice to be able to set a reminder to visit it each day."*
Unexpected Life Circumstances as Barrier to Engagement	*"The timing was in sync with becoming a solo parent and the death of a best friend, so this fall I didn't have additional time to focus."*
Need for More Flexibility in Utilization	*“Sometimes the information included in each trek didn't match the time of day that I did it and there wasn't enough preparatory time to work on it effectively.”* *“Allowed to only take small steps at a time.”*
Need for An Activity Log or Progress Tracking	*"I don’t remember what I typed from day to day, so it would be nice if it reminded me the next day what my goal was."* *"I would like for it to reference what I typed as an intervention or behavior previously when it mentions it in a later session."*
Technical Glitches and App Performance Issues	*"It has been difficult because the videos in each trek are not loading—they play, but the video is black, and you can only hear the audio, so I have to have a separate computer or switch back and forth on my phone to YouTube to look up the video so I can watch it."*

Despite the barriers, parents who engaged with the *Treks* app significantly decreased from Time 1 to 2 in perceived total stress, *M(SD)*_pre_ = 106.83 (16.78) vs. *M(SD)*_post_ = 94.08 (22.25); *t* = 4.15, *p* < .01,* d* = 0.65, and increased in self-perceived parenting competence, *M(SD)*_pre_ = 62.25 (7.21) vs. *M(SD)*_post_ = 69.83 (7.03); *t* = -4.29, *p* < .01, *d* = 1.06, with these improvements representing medium to large observed effects. Parents’ responses on the PSI-4-SF revealed that two of the 12 participants (16.67%) showed reliable decreases in total parenting stress (*RCI* = -2.94; *RCI* = -2.94). Regarding participants’ sense of parenting competency on the PSOC, 50% of participants demonstrated a reliable increase in their own parenting competence (*RCI* = 2.15–2.79). On the ECBI, non-significant improvement in behavior problem intensity was observed, *M(SD)*_pre_ = 64.00 (5.64) vs. *M(SD)*_post_ = 60.92; *t* = 1.57, *p* = .071,* d* = .45. Given that not all children were clinically elevated on the ECBI pre-intervention, a follow-up exploratory analysis with the sample restricted only to those who exceeded the cutoff on the ECBI resulted in the observed effect sizes increasing to *d* = .65. Overall, 83% of families who successfully completed the *Treks* BPT app noted an improvement in at least one study outcome variable.

### Study 2 – Pilot RCT for Families of Autistic Youth

#### Participants and Procedures

Participants included 26 parents and their autistic children (*M*_*age*_ = 6.81, *SD* = 2.91). Parents needed to speak English and have private access to internet and videoconferencing (i.e., smartphone, tablet, computer), and report concerns related to at least one of the following categories of behavior difficulties in their child: tantrums, noncompliance, difficulties with transitions, and/or aggression. Eligible children were between 3–13 years of age with an ASD diagnosis given by a doctor, counselor, or other qualified treatment provider, who presented with receptive language skills equivalent to or greater than 18 months on the Peabody Picture Vocabulary Test, 5th Edition (PPVT-5; Dunn, 2019). Participants were allowed to be enrolled in treatment with stable medication or no medication prior to entry and were not asked to stop services while enrolled in the study. Because of the nature of the treatment and its focus on targeting disruptive behaviors present outside of a clinical setting, the study excluded children with severe suicidal or homicidal behaviors or severe medical conditions (i.e., seizures). To ensure participant experience across the two conditions was as similar as possible, participants in the CC condition still downloaded the Treks app but this was only to answer the daily check-in questions and to access a resource page (i.e., they did not have access to the 28 Treks and their content). As can be seen in Table 2, the sample represented predominately middle-to-upper class socioeconomic statuses based on family income and parental education. Participants in the Treks and CC groups did not significantly differ on any demographic variables (*p*s > .062; see Table 2).

At time 1, an initial screening consisting of a phone call with the parent to determine eligibility, go over the online consent form, and to identify target behavioral problems that are of the parent’s primary concern (e.g., aggression, tantrums, repetitive behaviors), and a telehealth session via Zoom with the parent and child to confirm the child’s ASD diagnosis using the rCARS2 Observation were conducted. At time 2, a one 1.5–2-h telehealth consultation session to overview evidence-based behavioral strategies, and random assignment to either 4 weeks of telehealth support with the *Treks* app (Treks group; *n* = 14) or to 4 weeks of access to a resource page as a consultation control condition (CC group; *n* = 12) that included resources to assist parents on the implementation of evidence-based behavioral strategies took place. The child was required to score above the clinical cutoff on either the Social Responsiveness Scale, 2nd Edition (Constantino & Gruber, 2012) or on the Social Communication Questionnaire, Lifetime (Rutter et al., 2003) and meet criteria for ASD on the rCARS2 Observation (Sanchez & Constantino, 2020) to be eligible for this study. Parents completed questionnaires in both groups to track progress (e.g., parent knowledge, parent competence, parent stress, child problem behaviors), at the time of consultation (Time 2) and again one month after consultation for both groups after they completed 4 weeks of either Treks or CC behavioral support (Time 3). Data were stored on REDCap (Harris et al., 2009). Participants were compensated for completing study measures at each timepoint. This study was approved by the Virginia Tech Institutional Review Board and was performed in accordance with the ethical standards laid down in the 1964 Declaration of Helsinki.

### Measures

#### PSI-4-SF (Abidin, 2012)

The PSI-4-SF (described in Study 1) was administered at all timepoints to measure change in parenting stress. The Cronbach’s alphas indicate excellent internal consistency of the PSI-4-SF at all three time-points (α = .91, .91, and .92, respectively).

#### PSOC (Johnston & Mash, 1989)

Parents completed the PSOC (described in Study 1) at all three timepoints to measure change in parenting-self efficacy as related to child problem behaviors over the course of the study. Fair to good internal consistency was observed for the satisfaction domain (α = .75, .70, and .68) and the efficacy domain (α = .76, .79, and .83) at all time points.

#### ECBI (Eyberg & Pincus, 1999)

The ECBI (described in Study 1) was administered at all three timepoints to measure change in child problem behaviors. Cronbach’s alphas indicate excellent internal consistency of the ECBI (α = .93, .93, and .94 at T1-T3, respectively).

#### ***Treks*** Telehealth Satisfaction Survey (TTSS)

Parents completed a questionnaire developed for this study, modified from the Telehealth Satisfaction Scale (Morgan et al., 2014), to obtain parental feedback following completion of the *Treks* app. The 10-item survey assessed the satisfaction of the app, ease of use, and appropriateness of the mobile app model for addressing the target behavior, and comfort with the app. Eight items are rated on a 4-point scale (ranging from *Poor* to *Excellent*), 1 item is rated on a 5-point scale (ranging from *Very Dissatisfied* to *Very Satisfied*), and 1 item asked participants to provide additional feedback or comments. This measure was modified for those in the CC group to ask questions about the satisfaction, ease of use, and appropriateness of the resource page (the phrase “Treks mobile app” was replaced with “Treks mobile resources”). The TTSS was administered at Time 3.

#### Acceptability, Appropriateness, and Feasibility of Implementation

The AIM, IAM, and FIM (described in Study 1) were administered to parents at Time 3. In the current study, internal consistency was excellent for the AIM (α = .93), IAM (α = .96), and FIM (α = .95).

### Data Analytic Plan

**Fig. 1:**
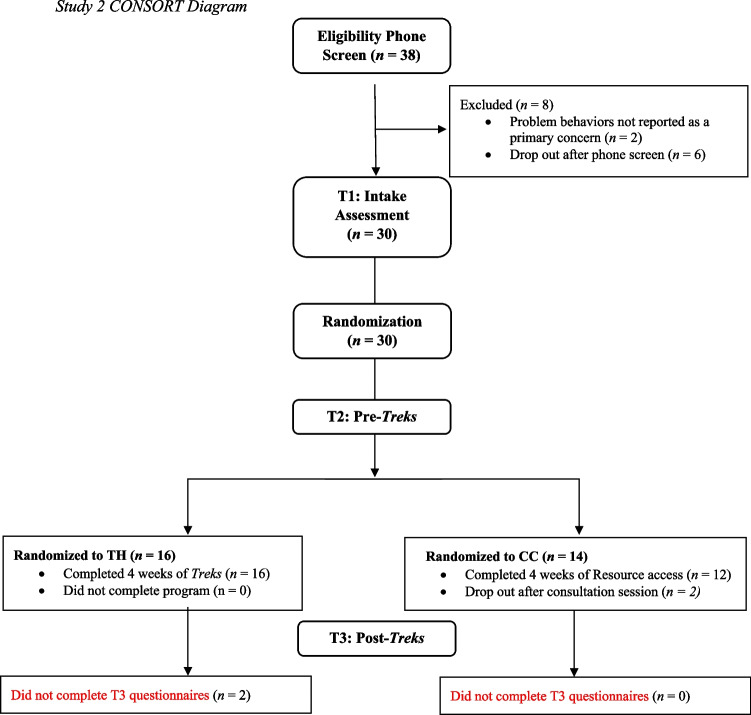
Study 2 CONSORT Diagram*.* Note. CC = Consultation Control condition. Due to us using paired samples *t*-tests for analyses, the 2 participants in the Treks condition who did not complete T3 questionnaires and the 2 participants in the CC condition who dropped out after consultation were not included in analyses resulting in the final sample of 14 in the Treks condition and 12 in the CC condition

First, to assess the feasibility of the *Treks* app, compliance with the study protocol and ease of use and appropriateness of the *Treks* app were assessed, including reasons for declining participation (e.g., parents are without internet access or a mobile device), parent completion of the full protocol, and reason for dropout. Percentages were calculated for participants who were eligible but declined to enroll, declined due to lack of internet access or a mobile device, and completed the consultation session but did not finish the *Treks* app. A CONSORT (Consolidated Standards of Reporting Trials; Schulz et al., 2010) diagram is included to demonstrate the flow from recruitment to participation and completion of the study (Fig. 1). As was done in Study 1, to test the hypothesis of acceptability, appropriateness, and feasibility of the app, scores from the AIM, IAM, and FIM were calculated to determine an overall mean score.

Next, we examined whether participants improved in parent competence (PSOC), stress (PSI-4-SF), and child problem behaviors (ECBI) compared to the CC group. As a preferred alternative to a power analysis, Hedeker et al. (1999) noted the following sample size estimations as applicable for longitudinal designs with two groups while accounting for attrition: To detect a large effect, the sample size required at Time 1 ranges from 12 to 15 for attrition rates of 0.00 to 0.10 and large effects, a sample size of 31 to 38 is required to detect a medium effect, and a sample size of 192 to 237 is required to detect a small effect. Thus, the current sample size (*n* = 26) for this study falls in the minimum range to detect a large effect between groups. As such, and since this was a pilot study, effect sizes were examined in addition to significance levels. Paired samples *t*-tests were used to examine parent stress (PSI-4-SF), competence (PSOC), and child problem behaviors (ECBI) from pre-*Treks* (Time 2) to post-*Treks* (Time 3). As with Study 1, individual level change in parent stress, competence, and child problem behaviors was also examined using RCI (Jacobson & Truax, 1991), using values ± 1.96 to infer statistically significant and meaningful change.

## Results

### Aim 1: Assessing Feasibility and Acceptability of Treks for Families of Autistic Children

As can be seen in Fig. 1, of the 30 families who were eligible and randomized, two participants (both in CC condition) did not use the online resources following the consultation session and two Treks participants did not complete the entire *Treks* app protocol and did not complete the post-treatment measures. Results on the TTSS demonstrated moderate to high satisfaction for participants in both the Treks and CC conditions. For the Treks condition, the mean rating of satisfaction for the *Treks* app was 31.79 (*SD* = 5.28; *Range* = 20–37); for the CC condition, the mean rating of satisfaction for the online resources was 33.83 (*SD* = 2.95*; Range* = 27–37). Twenty of the 26 participants (77%) rated each item as at least 3 or higher, with 6 participants rating some items a 2 (*Fair*), indicating moderate satisfaction (Treks = 4; CC = 2). Items that were given a score of 2 included “The quality of using the *Treks* mobile app or mobile resources” and “Your personal comfort in using the *Treks* mobile app or mobile resources.” None of the participants rated items on the TTSS as 1 (*Poor*). Further, participants provided qualitative data via a single item on this measure. Most participants provided comments on this item, including appreciation of the resources offered but also some frustration with technical issues faced while using the app (i.e., three participants were kicked off the app after completing the first week and needed to restart). This feedback is organized in Table 4.

**Table 4:** Qualitative Feedback for Study 2

Topic of feedback	Exemplar quotes
*Treks Group*	
*App Structure & Content*	“I really enjoyed this app. The only thing I would change is being able to share the videos (with other caregivers) and to be able to go back and re watch the videos. Also, allowing other caregivers to complete the treks as well so everyone is on the same page. I also think a weekly check-in with a clinician could be beneficial.” “I liked the concept of using an app to help guide parents in assessing their child's behavior. I think it would be helpful if more parents had that, especially in the early stages of being diagnosed.” “Very good! I liked the reminders and videos plus personalized tips and advice.” “The app is a bit too simplistic, and many examples were not applicable to our case.”
*Activities & Implementation*	“I enjoyed that everything was broken down in manageable assignments/tasks which made it feel less overwhelming when learning and implementing strategies.” “Useful and manageable program that was a huge help.” “I love this study. It helped a lot with my son's hitting. Thank you so much!” “Overall, I learned and implemented lots of new strategies.”
*Technical Issues*	“I found the app to be great except for being knocked and having to start at the beginning, that was a bit frustrating when I had to start over.” “I enjoyed using the mobile app. However, it did not work properly for me. It took me back to the beginning and I wasn't able to move forward from the point I was at.” “The embedded videos failed during our run, but content was well done and helpful!”
*Consultation Control Group*	
*App Structure & Mobile Resources*	“I was genuinely surprised how much I came to enjoy the app. Very well done. The clinicians are amazing. We feel enormously blessed to live in a community with this resource.” “Identifying the function and motivation to be consistent with consequences were the most valuable pieces of this study for me.” “I wish the app instructed me to "do" and activity every day, such as read an article or try XYZ intervention. Some days I'm just so overwhelmed with what's going on with my kiddo, having the prompt to try an intervention or activity may be just what I need to push through the day without feeling completely defeated.”
*Consultation Session*	“Everyone was very nice and informative. They answered all my questions, and I knew what I was going into.” “I appreciated the resources and thorough overview during the zoom session.” “The app that I had didn't really seem to go much with what they discussed in the consultation sessions on zoom. I got way more out of their presentation on the ABCs of behavior than the app.”

Similarly, among the Treks group scores from the AIM, IAM, and FIM questionnaires indicated that the *Treks* app was acceptable (*M* = 16.21, *SD* = 3.41; Range = 11–20), appropriate (*M* = 15.86, *SD* = 2.91, Range = 10–20), and feasible (*M* = 16.14, *SD* = 2.80, Range = 12–20) to address behavioral concerns in autistic children. Thirteen of the 14 participants rated each item as at least 3 or above for all of the questionnaires, with one participant providing a score of 2 (Disagree) on four items (“The *Treks* app is appealing to me”, “I like the *Treks* app”, “The *Treks* app seems fitting”, and “The *Treks* app seems applicable”.

### Aim 2: Assessing the Efficacy of Treks

Paired samples *t*-tests revealed a significant and large decrease from pre- to post-*Treks* in the Treks group on the PSI-4-SF, *M(SD)*_pre_ = 60.29(8.99) vs *M(SD)*_post_ = 54.86 (8.45)*, t* = 3.39, *p* = .005, *d* = .91. On the PSI-4-SF, 3 participants in the Treks group (21.4%) showed reliable decreases in total parenting stress (*RCI* = -2.141; *RCI* = -2.498; *RCI* = -2.855). Similarly, the parent sense of competence also significantly and largely improved in the Treks group, *M(SD)*_pre_ = 61.71 (9.51) vs *M(SD)*_post_ = 66.14 (9.21)*, t* = -3.50, *p* = .004, *d* = .94. Two participants in the Treks group (14.3%) showed reliable increases in parent competence (*RCI* = 3.212; *RCI* = 3.925). Lastly, a significant and medium-sized decrease was observed for the ECBI, *M(SD)*_pre_ = 140.29 (32.05) *vs. M(SD)*_post_ = 127.86 (36.62)*, t* = 2.32, *p* = .037, *d* = .62. On the ECBI, 7 participants in the Treks group (50%) showed reliable decreases in child behavior problems (*RCI* = -8.921; *RCI* = -7.851; *RCI* = -4.818; *RCI* = -5.353; *RCI* = -2.498; *RCI* = -2.498; *RCI* = -3.747), while 1 participant (7.1%; *RCI* = 2.676) showed a reliable increase in child behavior problems.

When examining the outcomes for the CC group from Time 2 to Time 3, results indicated no significant changes in main outcomes, *p*s > .078. However, there were small to medium decreases in parenting stress *(d* = .409) and child behavior problems (*d* = .499) and large improvements in parent competence *(d* = .561).

## Discussion

The current study examined the feasibility, acceptability, and preliminary efficacy of using the *Treks* BPT mobile app either as a standalone intervention (Study 1, parents of children with behavioral problems regardless of diagnosis) or as part of a technology-supplemented consultation model (Study 2, parents of autistic children) to support parents of children with behavioral difficulties. Results from both studies support the feasibility of the *Treks* BPT app for parents from middle to high socioeconomic backgrounds based on attrition, compliance, acceptability, ease of use, and appropriateness of the app; however, parents also provided feedback regarding issues with accessing the app and the potential of supplementing the app with consultations or check-ins. Both studies suggest preliminary evidence of benefits on parent outcomes (parenting stress and competence); however, significant improvements in child behavior problems only occurred for those who received the consultation session prior to utilizing *Treks* (Study 2; this effect was small and approaching medium in effect size, but non-significant for Study 1). These findings, their implications, and important areas for future mobile app interventions and research are now discussed.

### Feasibility and Acceptability of a Mobile BPT App

The *Treks* app and the *Treks*-supplemented consultation model were found to be feasible for families of children with behavior problems, regardless of presenting diagnosis, including for families of autistic children. This was evidenced by high compliance and low attrition, in addition to high levels of parental satisfaction ratings on the TTSS measure (Study 2), and moderate to high ratings of acceptability, appropriateness, and feasibility of the app on the AIM, IAM, and FIM scales (Study 1 and 2). Interestingly, based on the satisfaction measure, individuals in the CC group of study 2 appeared to have a more positive experience from the consultation session than the Treks group. One potential explanation is that these parents did not have the guided activities embedded in the *Treks* app itself to compare the consultation session to. This possibility is also supported by the results of Vismara et al. (2013), in which live videoconferencing was provided as a supplement to a self-guided parent training website, a platform that has a similar purpose to the *Treks* app, and found the weekly video conferencing sessions and website video modules to be the most helpful aspects of the program. It is also possible that the technical difficulties experienced by some *Treks* users in the Treks group lowered their satisfaction.

In terms of qualitative feedback on satisfaction with the Treks app, many participants in both studies provided positive feedback on the structure and content of the app (e.g., enjoying the videos and activities the *Treks* app had them do). Additionally, participants provided helpful suggestions for improvements to the app (listed in Tables 3 and 4). For example, parents suggested that allowing multiple caregivers to use the app so other family members in the home could be consistent in terms of implementing specific behavior management strategies, and that daily reminders in the app to complete their *Trek* lesson or weekly brief check-ins with clinicians either via a phone call, text message, or video call would have been helpful. Parents also noted in both studies that greater flexibility in utilization of the app, such that they can make up for missed days on days when they have time, and having a log to be able to reference previously typed responses during later treks would have been beneficial. Finally, several participants reported concerns regarding technical issues. Specifically, six participants in Study 1 (30%) and three participants in the Treks condition of Study 2 (21%) faced some technical difficulties (e.g., having to restart the *Treks* app after already completing a portion of the program) with the app, highlighting the importance of troubleshooting thoroughly when implementing a new app. Further, towards the end of the study one participant reported that some videos in the app did not run (likely because they were YouTube links that expired), so future iterations of this app or other mobile platforms would benefit from more reliable BPT training videos developed in-house.

### Preliminary Efficacy of a Mobile BPT App

The preliminary efficacy of the *Treks* app was partially supported across the two studies. Specifically, in Study 1, *Treks* was found to significantly decrease parent stress and improve parent competence among parents of children with behavioral problems (with moderate to large effects), but did not significantly improve parent-report of child behavior problems (despite near moderate effects for problem intensity). In contrast, in Study 2, those who utilized *Treks* (compared to a one-session telehealth consultation without the support of the *Treks* app) evidenced significant improvements in parent stress and competence. Since Study 1 did not require formal diagnoses and instead only required parent-report of behavior problems to be enrolled (with only 30% of participants having children with prior diagnoses of ASD, ADHD, and/or ODD), we believe the difference in findings may be driven by the lower frequency and intensity of behavior problems for the Study 1 sample compared to the Study 2 sample. Supporting this notion, follow-up exploratory analyses indicated that if the Study 1 sample was restricted only to those who exceeded the cutoff on the ECBI, observed effect sizes increased to *d* = 0.40 and .65. Alternatively, it is possible that the addition of a consultation session that primed parents on the behavioral principles enhanced the benefits of the subsequent intervention.

Importantly, results from Study 2 participants in the CC group showed that the consultation and access to online resources only model did not result in significant improvements across any outcome measures. This suggests that having an app deliver evidence-based strategies on a semi-regular basis over a month (or more) results in better outcomes than simply giving parents access to these sample resources/videos simultaneously. Notably, there were still small to moderate effects for the CC group, suggesting that access to consultation plus publicly available and organized evidence-based online resources may still provide some benefits to families who wouldn’t otherwise be able to access evidence-based treatment.

The *Treks* app incorporates activities for parents to engage in during the week, helps them set up a reward system, and provides words of encouragement to give positive reinforcement throughout the program, motivational aspects that are lacking from an online resource page. Given that only some of the participants in both studies demonstrated meaningful change in outcome variables on an individual level, as evidenced by their RCI, exploring the utility of these interventions as part of a stepped-care model is an important area for future research. Specifically, in such an approach, participants who do not benefit from the less intensive and costly approach (i.e., do not display meaningful change from the online resources and/or *Treks* app) get moved to consultation sessions and/or clinician-delivered BPT, which are more costly and time/resource-intensive.

Collectively, findings add to the limited prior research (Feil et al., 2018; Lee et al., 2024) on the efficacy of mobile BPT apps by extending it to a larger child age range and with more robust measures of parent and child improvement (i.e., assessing RCI). Additionally, they extend prior research showing benefits of video consultation sessions for parents of autistic children (Vismara et al., 2016), by suggesting that a single consultation session may be adequate, if then supplemented either with online resources or the mobile app.

### Limitations and Future Directions

These findings should be interpreted with several important limitations in mind. First, despite this study trying to provide an accessible intervention for families in rural communities, an eligibility criterion was that all participants had access to reliable technology by means of a stable internet connection and an Apple or Android cell phone device. In many remote or rural areas, this is not always feasible. Thus, future directions should examine alternate ways to access telehealth resources or apps using alternative devices (e.g., interactive websites, tablets). Second, most of the participants in both studies identified as White and represented middle to upper-middle class socioeconomic backgrounds, with participants needing to be fluent in English. Future research should make efforts to recruit a more representative sample of rural communities, including those from lower socioeconomic statuses and with access to less resources, and should investigate how to adapt these resources to various cultures and language levels to better disseminate evidence-based strategies to parents from various marginalized and underserved communities, as well as if cultural adaptations (beyond language translation) may be needed.

Further, the sample size of both studies alone (or even combined for Study 1 and the Treks subsample of Study 2), was not large enough to run more complex analyses to assess efficacy and whether variables such as receptive language, age, or socioeconomic status may influence treatment outcomes. It will be critical for future studies to explore such potential moderators of treatment outcomes in a larger, more diverse sample. Our two studies also were collected through different means, with Study 1 representing a broader community sample, with recruitment largely coming from community postings and contacting families on clinic waitlists, relative to Study 2 which was a clinical sample of families who already had received a diagnostic assessment (and in some cases, intervention), with formal ASD diagnoses. Examining predictors of variance in parent and child outcomes, including through person-centered analytical approaches, is an important area for future research. Finally, all of our outcomes in this initial pilot feasibility study were based on parent-report. Since parents were aware of their study condition, it is possible that parents may have experienced expectancy effects (), where their ratings reflect their expectations for treatment outcomes rather than actual improvements. As such, it will be critical for future research to involve multi-rater and/or multi-method (e.g., observations of child behavior) assessment of outcomes.

## Conclusions

The objective of the current study was to investigate the feasibility, acceptability, and preliminary efficacy of a mobile app BPT intervention to support parents of children with behavioral difficulties transdiagnostically in a community sample and specifically for parents of autistic children. Findings revealed adequate feasibility and acceptability, and variable but promising parent and child outcomes for this mobile app and provides preliminary support for a mobile app being used as a standalone intervention or integrated into other clinician-delivered care (such as the consultation model). Future refinement based on user feedback, and assessment of mobile BPT apps is needed, particularly as part of a stepped-care model, and with more socioeconomically and racially-ethnically diverse and underserved populations. Such research has the potential to improve parent and child outcomes and further validate this more accessible and cost-effective intervention.

## Data Availability

Data is available by request to the corresponding author.
